# Detection of NLRP3, ASC, and Caspase-1 in serum and cerebrospinal fluid of traumatic brain injury patients: implications for short-term prognosis

**DOI:** 10.1007/s13760-025-02761-8

**Published:** 2025-03-11

**Authors:** Adilijiang Aihemaitiniyazi, Kuo Ma, Jinhui Xu, Hua Chen, Xianglu Liu, Jilin Li

**Affiliations:** 1 Department of Neurosurgery, Qingpu Branch of Zhongshan Hospital, Fudan University, Shanghai, China; 2 Department of Clinical Laboratory, Qingpu Branch of Zhongshan Hospital, Fudan University, Shanghai, China

**Keywords:** Traumatic brain injury, NLRP3 inflammasome, Biomarkers, Neuroinflammation, Prognosis

## Abstract

**Objectives:**

Traumatic brain injury (TBI) is a critical public health issue with high mortality and disability rates. Current diagnostic tools lack sensitivity and specificity, under-scoring the need for novel biomarkers. This study aimed to evaluate the clinical utility of NLRP3, ASC, and Caspase-1 as biomarkers for assessing TBI severity and prognosis.

**Methods:**

A prospective cohort of 89 patients with moderate-to-severe TBI was studied. Blood and cerebrospinal fluid (CSF) samples were collected for four consecutive days post-injury. Levels of NLRP3, ASC, and Caspase-1 were measured using enzyme-linked immunosorbent as-say (ELISA). Statistical analyses, including ROC curve analysis, were conducted to assess their predictive performance.

**Results:**

NLRP3, ASC, and Caspase-1 levels in both serum and CSF were significantly elevated in TBI patients, with higher levels correlating with greater injury severity. ROC analysis revealed that CSF biomarkers, particularly NLRP3, demonstrated superior predictive value. CSF NLRP3 levels on days 1, 2, and 4 had AUC values of 0.9871, 0.9466, and 0.8967, respectively. Dynamic changes in these biomarkers over time provided insights into disease progression and prognosis. Serum markers, while less predictive than CSF, were also effective for assessing injury severity.

**Conclusions:**

NLRP3, ASC, and Caspase-1 are promising biomarkers for evaluating TBI severity and predicting outcomes. Their dynamic monitoring may improve clinical management and in-form therapeutic strategies. Future research should validate these findings in larger cohorts and explore interventions targeting these inflammatory pathways.

## Introduction

Traumatic brain injury (TBI), caused by external trauma, is a form of brain tissue damage and is one of the leading causes of disability and mortality among children and adolescents globally [1, 2]. TBI is a significant global health issue and a leading cause of death. A large-scale study across 16 European countries found a TBI hospitalization rate of 287.2 per 100,000 individuals, with a mortality rate of 11.7% [3]. In China, statistical reports indicate an annual TBI mortality rate of 12.99 per 100,000 people [4]. Moreover, unlike in Western countries where TBI primarily affects the elderly, in China, the majority of TBI patients are middle-aged or younger adults. This demographic difference presents unique medical challenges, as the demand for surgical intervention is significantly higher in this population [5]. Consequently, this places immense pressure on healthcare resources and results in substantial social and economic burdens [6].

Although the mortality rate of TBI has declined in recent years due to the widespread implementation of evidence-based TBI guidelines and advancements in critical care, a significant number of survivors continue to suffer from various neurological impairments caused by neuronal damage [1, 7]. These include deficits in language, learning, emotional regulation, cognitive behavior, and even mental illnesses, making the diagnosis and treatment of TBI a major challenge for healthcare professionals [8, 9]. There is an urgent clinical need to develop more specific and sensitive molecular biomarkers, as well as effective therapeutic targets for TBI, to enhance diagnostic and treatment outcomes [10]. Early identification of biomarkers that can predict the severity and prognosis of TBI is crucial for improving outcomes and guiding therapeutic strategies [11].

Currently, the U.S. Food and Drug Administration (FDA) has approved biomarkers such as Ubiquitin C-terminal Hydrolase L1 (UCH-L1) and Glial Fibrillary Acidic Protein (GFAP) for clinical use in TBI diagnosis [12]. These biomarkers, measured in serum, can help assess whether a mild TBI patient requires further imaging. Studies have demonstrated that the levels of UCH-L1 and GFAP gradually increase in mild TBI patients following injury, peaking at 6 and 12 h post-injury, respectively, and returning to baseline after approximately 48 h [13]. These markers are useful for distinguishing between mild and moderate cases and determining the need for additional diagnostic interventions [14]. Additionally, it would be of interest to investigate how inflammatory markers such as NLRP3, ASC, and Caspase-1 compare to GFAP and UCH-L1 as potential biomarkers for TBI diagnosis. These markers are involved in the inflammasome pathway, which has been implicated in TBI-related neuroinflammation, and could provide complementary or even superior diagnostic value when assessing the severity of TBI.

Furthermore, there is growing interest in identifying blood-based markers related to the inflammatory response, which may not only provide more insights into the patho-physiology of TBI but also help in assessing disease severity and predicting long-term outcomes. TBI primarily induces cell injury via mechanisms like excitotoxicity, neuroinflammation, oxidative stress, and cytokine dysregulation [12–14]. Among these, the NLRP3 inflammasome, a key component of the innate immune response, plays a critical role in driving neuroinflammation after brain injury [15]. The inflammasome is activated by damage-associated molecular patterns (DAMPs), leading to the activation of caspase-1 and the subsequent cleavage and release of pro-inflammatory cytokines such as IL-1β and IL-18. This activation also triggers proptosis, a form of programmed cell death that ex-acerbates brain injury and inflammation [6].

Animal models of TBI have provided critical insights into the role of the NLRP3 inflammasome in the pathophysiology of brain injury. Studies in rats have shown that the expression of NLRP3, ASC, and caspase-1 is significantly increased in cortical tissue after TBI, with NLRP3 levels peaking at 6 h post-injury [16]. These findings are directly correlated with the severity and progression of brain injury. Additionally, pharmaco-logical inhibition of the NLRP3 inflammasome using agents such as propofol, telmisartan, MCC950, or rapamycin, or conditional knockout of NLRP3, significantly reduces the inflammation induced brain damage, providing evidence for the therapeutic potential of targeting NLRP3 in TBI [17–21]. These preclinical results highlight the promise of NLRP3 inflammasome inhibitors as a novel therapeutic strategy for TBI.

However, clinical research on the expression and role of NLRP3 inflammasomes in human TBI is still relatively limited. Some studies suggest that the expression levels of NLRP3 inflammasome components such as mRNA or protein are elevated in the blood of TBI patients, and that these levels correlate with injury severity [22, 23]. Based on these findings, we hypothesize that the expression of the NLRP3 inflammasome in the blood and cerebrospinal fluid (CSF) of TBI patients is directly related to the severity of the injury and dynamically changes with the progression of the disease and response to treatment. This dynamic expression could serve as a valuable predictor for patient prognosis, particularly for evaluating recovery and anticipating potential complications following TBI. These biomarkers hold significant potential for improving clinical management, as they could assist in predicting outcomes, tailoring individualized treatment plans, and enabling early intervention strategies.

This study aims to explore the potential clinical applications of NLRP3, ASC, and Caspase-1 levels as biomarkers for assessing disease severity and predicting prognosis in TBI patients. Our findings could contribute to the development of more effective diagnostic and therapeutic approaches for TBI.

## Materials and methods

### Data collection methods

This study has been approved by the Ethics Committee of the Zhongshan Hospital Qingpu Branch, Fudan University. The study subjects are patients with TBI who were hospitalized at the Zhongshan Hospital Qingpu Branch, Fudan University, from January 1, 2023, to June 30, 2024. The inclusion and exclusion criteria are as follows:

Inclusion Criteria:

- Age ≥ 18 years with a confirmed diagnosis of acute TBI;
- Chinese citizens;
- Ability to provide informed consent personally or through a legal representative;
- Willingness to undergo multiple time-point blood tests and/or CSF tests (limited to patients requiring lumbar puncture for clinical purposes or those voluntarily agreeing to lumbar puncture) and to complete follow-up with comprehensive data.

Exclusion Criteria:

- A history of one or more TBI within the past year, or patients with trauma exceeding 24 h upon admission.
- Presence of severe systemic diseases (such as severe infections, uncontrolled autoimmune diseases, chronic alcoholism, drug abuse, or active metastatic ma-lignant tumors) that may affect inflammatory responses.
- Severe kidney disease or metabolic disorders leading to protein metabolism abnormalities.
- Use of high-dose systemic corticosteroids within 14 days prior to enrollment.
- Patients with conflicts of interest related to the study.
- Patients who fail screening or voluntarily withdraw from the study.
- Patients who cannot meet sampling requirements due to large volume blood transfusions (e.g., transfusion of > 1 times the patient's blood volume in a single session, or transfusion of > 1/2 blood volume within 1 h, or transfusion rate > 1.5 mL/(kg·min)).
- Patients who do not complete at least three tests due to poor compliance or dete-rioration of condition (e.g., severe anemia or death).
- Inability to complete at least three tests due to poor compliance or worsening condition, such as severe anemia or death.

### TBI group classification

According to the Glasgow Coma Scale (GCS), the severity of TBI was categorized as moderate (score 9–12) and severe (score 3–8) [24]. Following injury, patients underwent a 3-month follow-up period, during which their prognosis was assessed using the Glasgow Outcome Scale (GOS). At the end of the follow-up, a GOS score > 3 was considered as a good prognosis (good prognosis group), while a score ≤ 3 was considered as a poor prognosis (poor prognosis group). Specifically, a GOS score of 5 indicates good recovery with near-normal daily functioning, although some minor deficits may remain; 4 indicates mild disability with the ability to live independently and work under supervision; 3 indicates severe disability, awake but requiring daily care; 2 indicates a vegetative state with minimal response (e.g., following sleep/wake cycles, eye-opening); and 1 indicates death. CSF and fasting serum samples were collected from patients on days 1, 2, 3, and 4 following injury for the measurement of NLRP3 levels in both CSF and serum.

### Sample collection

Peripheral blood samples were collected from patients with moderate and severe TBI for four consecutive days post-injury. CSF samples were obtained from all participants. Severe patients and the majority of moderate patients underwent surgical intervention with ventricular drainage, while the remaining patients provided informed consent for lumbar puncture. These two approaches ensured successful CSF collection from all enrolled subjects.

#### Serum processing

Peripheral blood was collected in sterile, pyrogen-free tubes without anticoagulants to avoid cellular stimulation. After clotting at room temperature for 30 min, samples were centrifuged at 3000 × g for 10 min to separate serum. The supernatant was aliquoted and stored at −80 °C until analysis to prevent repeated freeze–thaw cycles.

#### CSF processing

CSF samples obtained via ventricular drainage or lumbar puncture were immediately centrifuged at 3000 × g for 10 min to remove cellular debris. The supernatant was aliquoted and stored at −80 °C until further analysis.

### Enzyme-Linked Immunosorbent Assay (ELISA)

ELISA testing was performed using the following commercially available kits:

NLRP3: Human NLRP3 ELISA Kit (MM-2198H1, MyBioSource, San Diego, CA, USA; 96-well plate),ASC: Human ASC ELISA Kit (MM-61069H1, MyBioSource, San Diego, CA, USA; 96-well plate),Caspase-1: Human Caspase-1 ELISA Kit (MM-13398H1, MyBioSource, San Diego, CA, USA; 96-well plate).

The assays were conducted strictly according to the manufacturer’s instructions:

- Capture Antibody Binding: Standards and samples (100 µL) were added to the antibody-precoated wells and incubated at 37 °C for 2 h.
- Detection Antibody Binding: After three washes with PBS-T, biotin-conjugated detection antibodies (provided in each kit) were added and incubated at 37 °C for 1 h.
- Signal Amplification: Following another washing step, streptavidin–horseradish peroxidase (HRP) conjugate was added and incubated at 37 °C for 1 h, forming a "capture antibody-target protein-biotinylated detection antibody-streptavidin-HRP" complex.
- Color Development and Quantification: Tetramethylbenzidine (TMB) substrate was added and incubated for 30 min at room temperature. The reaction was terminated with stop solution, and the absorbance was measured at 450 nm with a reference wavelength of 540 nm using a microplate reader. Concentrations of NLRP3, ASC, and Caspase-1 were calculated based on the standard curve.

### Statistical analysis

Statistical analyses were performed using GraphPad Prism (GraphPad Software, La Jolla, CA) and SPSS version 23 (IBM Corp, Armonk, NY). Categorical demographic variables were compared using Fisher’s exact test, while continuous variables were analyzed using Student’s t-test. For longitudinal analysis, the temporal trends of three biomarkers (NLRP3, ASC, and Caspase-1) across four time points were assessed using repeated-measures ANOVA followed by post hoc Bonferroni correction for normally distributed data. For non-normally distributed data, the Kruskal–Wallis test with Dunn’s multiple comparisons test was applied. To further investigate the correlation between biomarker levels and clinical variables, the predictive power of peak NLRP3 ASC and Caspase-1 levels for clinical outcomes was assessed using receiver operating characteristic (ROC) curve analysis, with the area under the curve (AUC) serving as an indicator of predictive accuracy. Continuous variables were presented as mean ± standard deviation (SD) and compared using either the two-sample t-test or the Mann–Whitney U test, depending on the data distribution. Categorical variables were expressed as frequencies (percentages) and analyzed using Pearson’s chi-squared test or Fisher’s exact test, based on the expected cell frequencies. A two-tailed P-value less than 0.05 was considered statistically significant.

## Results

### Study design and baseline characteristics

**Fig. 1:**
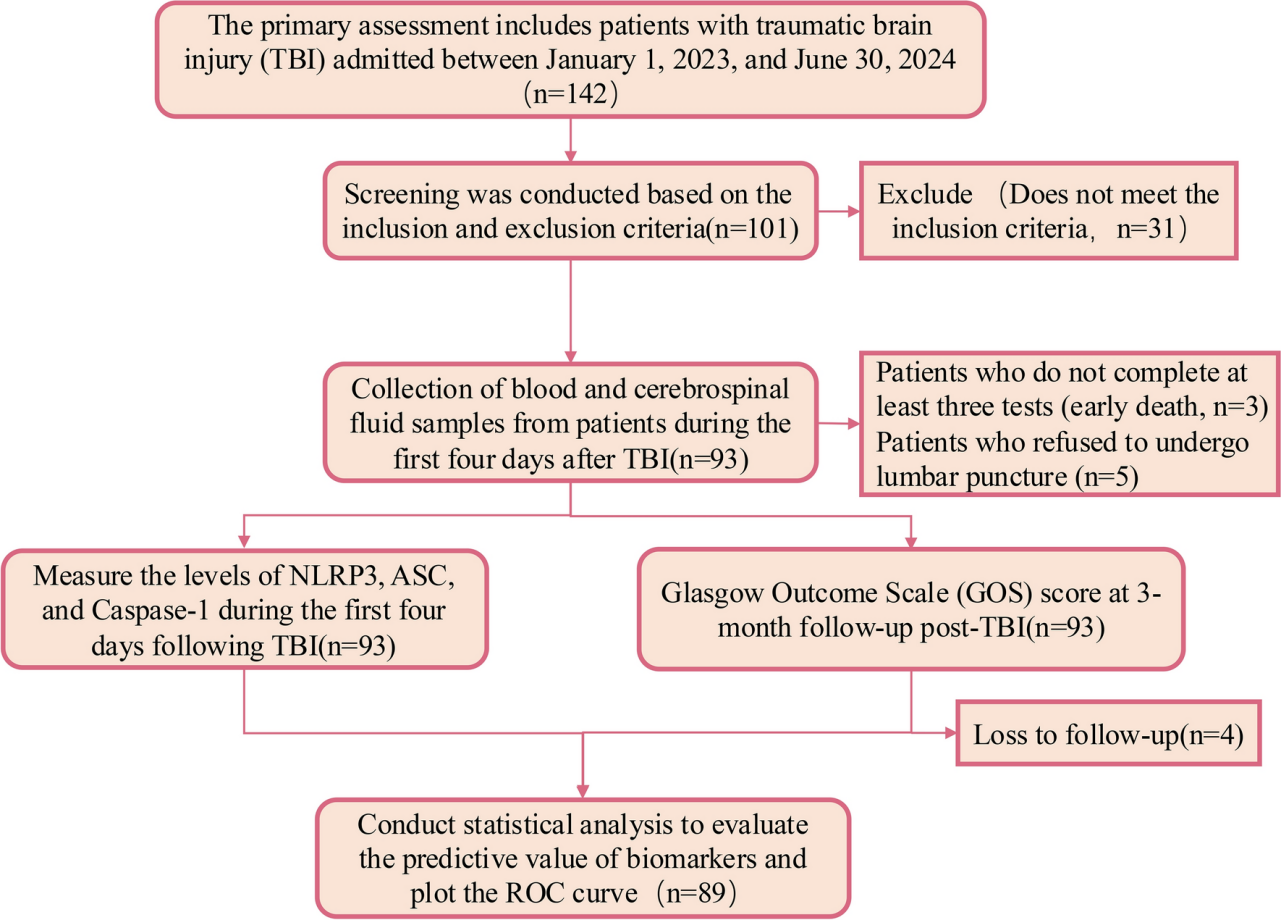
Flow chart of the entire research process

The study included patients with TBI who were hospitalized at the Zhongshan Hospital Qingpu Branch, Fudan University, between January 1, 2023, and June 30, 2024. A total of 142 patients were initially identified. After applying the inclusion and exclusion criteria, 101 patients met the eligibility requirements. During the early stages, three patients died, and five patients declined lumbar puncture. During the three-month follow-up period, four patients were lost to follow-up (as shown in Fig. 1).

Ultimately, 89 patients were included in the final analysis, with 72 (80.9%) male and 17 (19.1%) female participants, and the mean age was 54.2 ± 16.8 years. Patients were categorized into mild-to-moderate and severe groups based on the GCS scores at injury. The moderate severity group included 47 patients (40 males, 85.1%, and 7 females, 14.9%) with an average age of 54.4 ± 18.2 years and an average injury GCS score of 12.17 ± 1.93. The severe group comprised 42 patients (32 males, 76.2%, and 10 females, 23.8%) with an average age of 54.0 ± 15.6 years and an injury GCS score of 6.23 ± 1.37 (refer to Table 1).

**Table 1:** Comparative baseline clinical features in traumatic brain injury patients with different severity levels and prognoses

Characteristics	Good(n = 45)	poor (n = 44)	p-value
**Age (years)**			
< 60	39 (86.7%)	16 (36.4%)	0.002^*^
≥ 60	6 (13.3%)	28 (63.6%)	
**Gender (n, %)**			
Male	38 (84.4%)	34 (77.3%)	0.464
Female	7 (15.6%)	10 (22.7%)	
**Severity at injury**			
moderate	31 (68.9%)	16 (36.4%)	0.001^*^
Severe	14 (31.1%)	28 (63.6%)	
**Cause of injury**			
Accident	22 (48.9%)	18 (40.9%)	0.317
Falling	14 (31.1%)	16 (36.4%)	
Others	9 (20.0%)	10 (22.7%)	
**Hypertension**			
Yes	12 (26.7%)	23 (52.3%)	**0.007**
No	33 (73.3%)	21 (47.7%)	
**Cerebral contusion and laceration**			
Yes	9 (20.0%)	22 (50.0%)	0.001^*^
No	36 (80.0%)	22 (50.0%)	
**Intracerebral hematoma**			
Yes	5 (11.1%)	19 (43.2%)	0.001^*^
No	40 (88.9%)	25 (56.8%)	
**Epidural hematoma**			
Yes	3 (6.7%)	8 (18.2%)	0.175
No	42 (93.3%)	36 (81.8%)	

Note: Data are presented as frequencies with percentages (% indicating the proportion of each characteristic within the respective group). Comparisons between the two groups were performed using Fisher's exact test or Chi-square test, as appropriate. A two-sided *P* value of less than 0.05 was considered statistically significant. The terms "Poor" and "Good" refer to poor prognosis (GOS score ≤ 3) and good prognosis (GOS score > 3), respectively

Within the severe group, based on the Glasgow Outcome Scale (GOS) scores at a three-month post-injury follow-up, patients were further classified into groups with good and poor prognoses. The group with a good prognosis included 45 patients, having an average GOS score of 4.33 ± 0.49, while the poor prognosis group consisted of 44 patients, with an average GOS score of 1.82 ± 0.60. Analyzing the baseline data between the two groups, we found significant differences in aspects such as age, severity of TBI, occurrence of cerebral contusions and lacerations, intracranial hematoma.

### Temporal changes in NLRP3, ASC, and Caspase-1 levels in serum and CSF of TBI patients

**Fig. 2:**
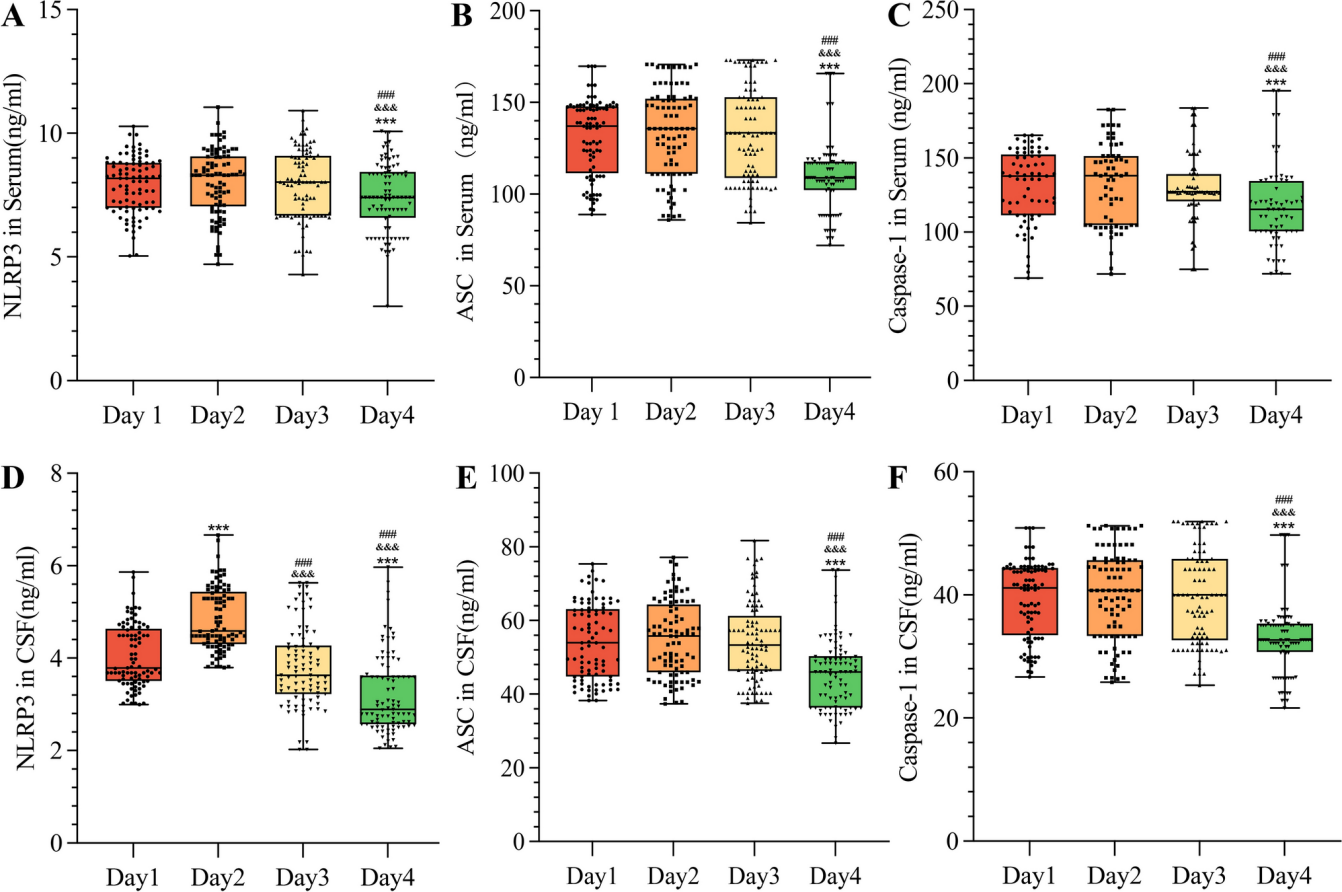
Temporal dynamics of NLRP3, ASC, and Caspase-1 levels in serum and CSF of patients with TBI (n = 89). Statistical analysis was conducted using the Kruskal–Wallis test followed by Dunn’s multiple comparisons test for intergroup differences. ***p < 0.001 compared with 1-day post-admission. &&&p < 0.001 compared with 2 days post-admission. ###p < 0.001 compared with 3 days post-admission

We conducted a systematic investigation of the temporal variations in NLRP3, ASC, and Caspase-1 levels in both serum and CSF during the initial four days post-injury, as illustrated in Fig. 2. The analysis revealed distinct dynamic patterns between different biomarkers and biological compartments. Notably, CSF concentrations of NLRP3 exhibited a characteristic biphasic response, reaching peak levels on day 2 post-injury before gradually declining to approach baseline values observed on day 1 by days 3–4.

In contrast, ASC and Caspase-1 displayed divergent profiles in the CSF compartment. These biomarkers failed to demonstrate the secondary elevation observed with NLRP3, instead showing progressive reductions that culminated in statistically significant decreases by day 4 compared to earlier time points (p < 0.05). The serum biomarker profiles differed substantially from CSF patterns, with no discernible early elevation phase observed for any of the measured analytes. However, similar to CSF findings, all serum biomarkers demonstrated significant reductions in concentration by day 4 relative to preceding measurements (p < 0.01).

### Differential levels of NLRP3, ASC, and Caspase-1 in serum and CSF between severe and moderate TBI groups

**Fig. 3:**
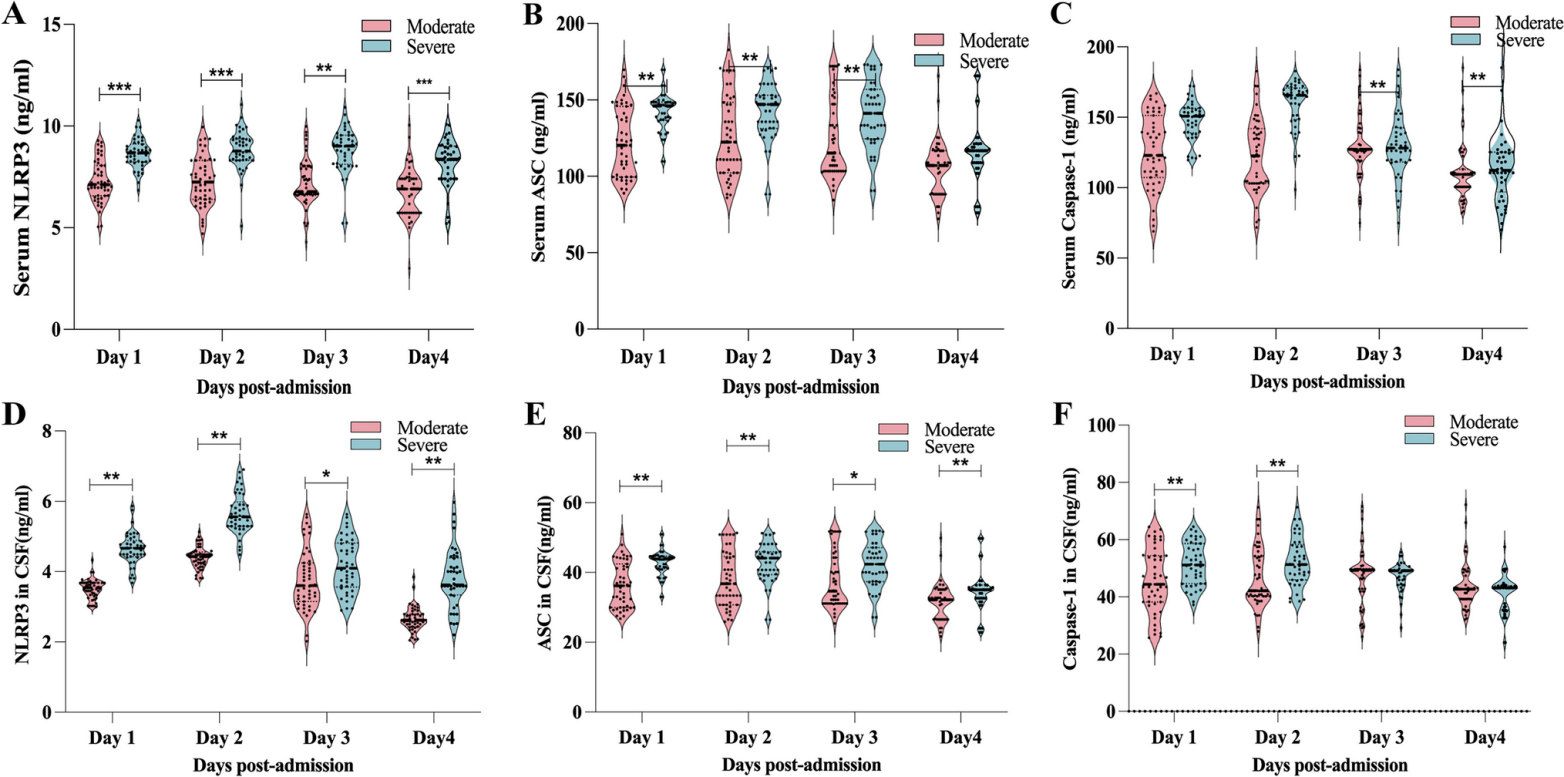
Serum (**A**, **B**, **C**) and CSF (**D**, **E**, **F**) levels of NLRP3, ASC, and Caspase-1 on days 1–4 post-injury in moderate (n = 47) and severe (n = 42)TBI groups. *p < 0.05, **p < 0.01, ***p < 0.001. This figure Comparative analysis of serum (**A**-**C**) and CSF (**D**-**F**) levels of NLRP3, ASC, and Caspase-1 between moderate (n = 47) and severe (n = 42) TBI groups across post-injury days 1–4. Statistical comparisons were performed using the Mann–Whitney U test)

The levels of NLRP3, ASC, and Caspase-1 in both serum and CSF showed significant differences between the severe TBI group and the moderate TBI group. Figure 3 illustrates the correlation between the levels of these markers in serum and CSF at days 1, 2, 3, and 4 post-injury and the severity of TBI.

Serum NLRP3 levels were significantly elevated in the severe TBI group compared to the moderate TBI group at all time points measured (p < 0.05, p1 = 0.0003, p2 = 0.0007, p3 = 0.00001, p4 = 0.0005). Serum ASC levels displayed significant differences on days 1, 2, and 3 (p < 0.05, p1 = 0.009, p2 = 0.00003, p3 = 0.003), but no significant difference was observed on day 4 (p > 0.05, p4 = 0.237). For serum Caspase-1, significant differences were detected on days 1 and 2 (p < 0.05, p1 = 0.0047, p2 = 0.0068), while no significant differences were observed on days 3 and 4 (p > 0.05, p3 = 0.9877, p4 = 0.4562).

In CSF, NLRP3 levels consistently showed significant differences between the two groups at all time points (p < 0.05, p1 = 0.0067, p2 = 0.0098, p3 = 0.045, p4 = 0.0076), suggesting its potential as a stable biomarker for TBI severity. CSF ASC levels were significantly different on days 1 and 2 (p < 0.05, p1 = 0.0065, p2 = 0.0074) but did not show significant differences on days 3 and 4 (p > 0.05, p3 = 0.397, p4 = 0.259). Similarly, CSF Caspase-1 levels exhibited significant differences on days 1, 2, and 3 (p < 0.05, p1 = 0.0098, p2 = 0.0034, p3 = 0.026), but no significant difference was observed on day 4 (p > 0.05).

These findings emphasize the potential of NLRP3 as a reliable biomarker in both serum and CSF for assessing TBI severity over time. In contrast, ASC and Caspase-1 appear to play more critical roles during the early stages of the inflammatory response. Furthermore, the observed changes in CSF markers may provide a more direct and accurate representation of central nervous system inflammation compared to serum markers, highlighting their importance in evaluating TBI severity.

### The predictive value of serum and CSF NLRP3, ASC, and Caspase-1 for TBI outcomes

**Fig. 4:**
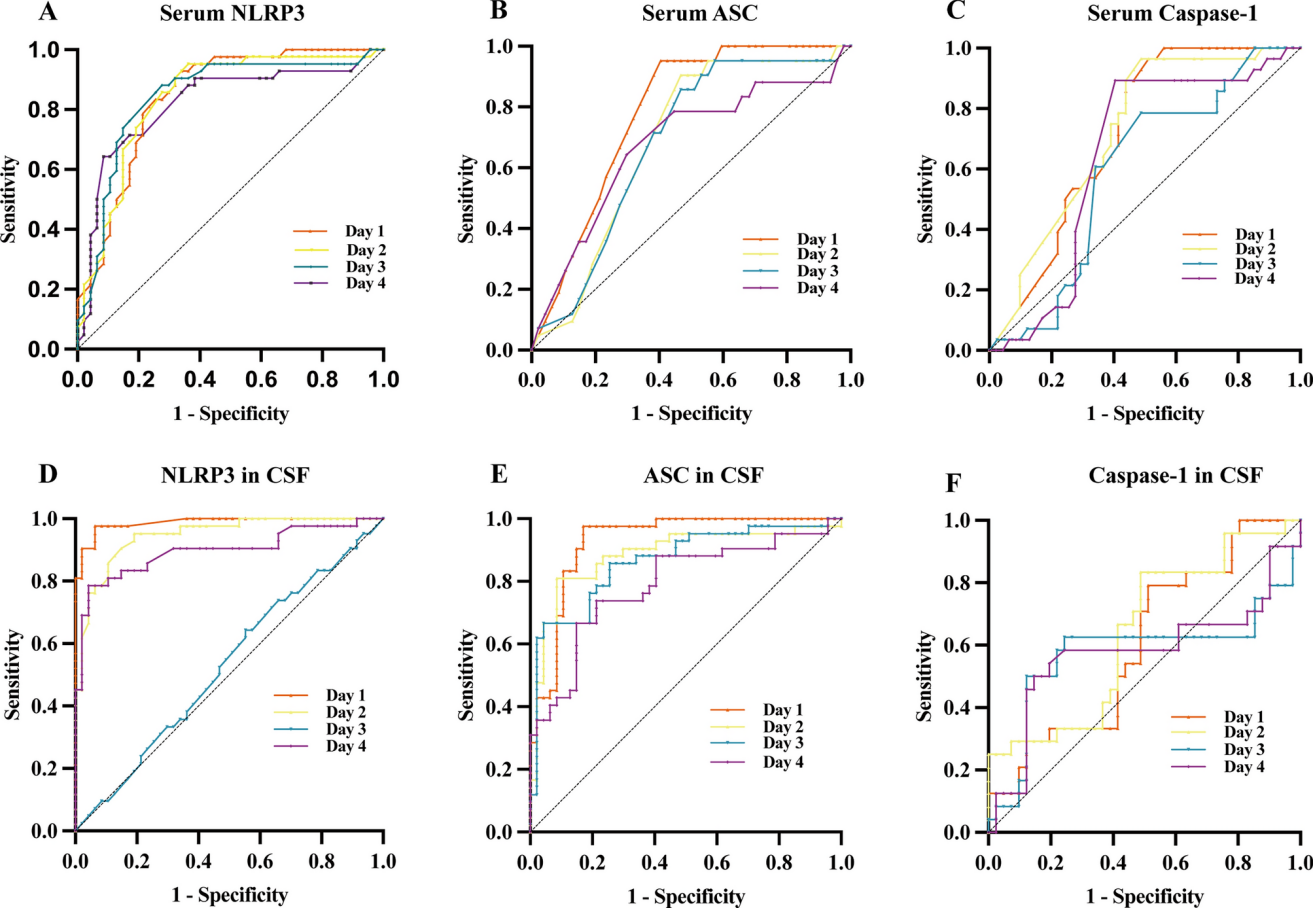
ROC analysis of the predictive values of NLRP3, ASC, and Caspase-1 levels in serum (**A**) and cerebrospinal fluid (CSF, **B**) at 1, 2, 3, and 4 days post-injury, assessing prognosis in patients with moderate-to-severe TBI brain injury

The ROC analysis demonstrated the predictive value of the inflammatory markers NLRP3, ASC, and Caspase-1 in TBI (Fig. 4). Both serum and CSF levels of NLRP3 exhibited high area under the curve (AUC) values. Notably, serum NLRP3 levels on days 1, 2, 3, and 4 showed the most significant predictive ability (Fig. 4A, with AUC values of 0.8445, 0.8402, 0.8442, and 0.8204, respectively). In the CSF, the NLRP3 levels on days 1, 2, and 4 demonstrated the highest predictive performance (AUC values of 0.9871, 0.9466, and 0.8967, respectively).

Serum ASC levels showed notable predictive value on day 1 (Fig. 4B, AUC = 0.7817), whereas CSF ASC levels exhibited better predictive performance, with AUC values of 0.9276, 0.886, 0.8637, and 0.7898 on days 1, 2, 3, and 4, respectively (Fig. 4E).

For Caspase-1, serum levels demonstrated higher predictive value on days 1 and 2 (Fig. 4C, AUC values of 0.7169 and 0.7239, respectively). In contrast, CSF Caspase-1 levels showed limited predictive performance, with AUC values of 0.9276, 0.886, 0.8637, and 0.7898 on days 1, 2, 3, and 4, respectively (Fig. 4F).

Table 2 presents the cutoff values, AUC, sensitivity, and specificity calculated based on the Youden index. The results indicate that both serum and CSF levels of NLRP3 have good predictive value and effectively distinguish between the two TBI patient groups. Additionally, ASC and Caspase-1 levels in CSF also demonstrated significant predictive potential.

**Table 2:** Predictive values in ROC analysis

	Cut-off value	AUC (95% CI)	p	Sensitivity	Specificity	Youden index
Serum NLRP3-1-day post-injury	7.58	0.8445 (0.7628–0.9261)	< 0.0001	0.9286	0.6596	0.59
Serum NLRP3-2-day post-injury	7.625	0.8402 (0.7546–0.9257)	< 0.0001	0.9524	0.6383	0.5907
Serum NLRP3-3-day post-injury	7.94	0.8442 (0.7568–0.9317)	< 0.0001	0.881	0.7234	0.6044
Serum NLRP3-4-day post-injury	8.31	0.8204 (0.7267–0.9142)	< 0.0001	0.6429	0.9149	0.5578
Serum ASC-1-day post-injury	123.80	0.7817 (0.6843–0.8790)	< 0.0001	0.9524	0.5957	0.5481
Serum ASC-2-day post-injury	124.00	0.6900 (0.5765–0.8035)	0.0021	0.9048	0.5319	0.4367
Serum ASC-3-day post-injury	118.80	0.6836 (0.5702–0.7970)	0.0029	0.8571	0.5319	0.389
Serum ASC-4-day post-injury	111.20	0.6773 (0.5625–0.7921)	0.0040	0.6429	0.7021	0.345
Serum Caspase-1–1-day post-injury	123.50	0.7169 (0.5974–0.8364)	0.0023	0.8929	0.561	0.4539
Serum Caspase-1–2-day post-injury	120.50	0.7239 (0.6044–0.8433)	0.0017	0.9643	0.5122	0.4765
Serum Caspase-1–3-day post-injury	126.80	0.5949 (0.4591–0.7308)	0.1829	0.7857	0.5122	0.2979
Serum Caspase-1–4-day post-injury	110.00	0.6463 (0.5162–0.7763)	0.0350	0.8929	0.5957	0.4886
NLRP3 in CSF-1-day post-injury	3.80	0.9871 (0.9702–1.000)	< 0.0001	0.9762	0.9362	0.9124
NLRP3 in CSF-2-day post-injury	4.85	0.9466 (0.9035–0.9896)	< 0.0001	0.9524	0.8085	0.7609
NLRP3 in CSF-3-day post-injury	3.47	0.5276 (0.4070–0.6482)	0.6542	0.6429	0.4468	0.0897
NLRP3 in CSF-4-day post-injury	3.20	0.8967 (0.8251–0.9682)	< 0.0001	0.7857	0.9574	0.7431
ASC in CSF-1-day post-injury	49.85	0.9276 (0.8728–0.9823)	< 0.0001	0.9762	0.8298	0.806
ASC in CSF-2-day post-injury	57.13	0.886 (0.8102–0.9619)	< 0.0001	0.8095	0.9149	0.7244
ASC in CSF-3-day post-injury	57.39	0.8637 (0.7852–0.9422)	< 0.0001	0.6667	0.9574	0.6241
ASC in CSF-4-day post-injury	46.34	0.7898 (0.6931–0.8865)	< 0.0001	0.7381	0.7872	0.5253
Caspase-1 in CSF-1-day post-injury	54.79	0.6075 (0.4641–0.7452)	0.1615	0.7917	0.4878	0.2795
Caspase-1 in CSF-2-day post-injury	53.95	0.6402 (0.4998–0.7807)	0.0607	0.8333	0.5122	0.3455
Caspase-1 in CSF-3-day post-injury	56.84	0.5722 (0.4023–0.7420)	0.3345	0.625	0.7561	0.3811
Caspase-1 in CSF-4-day post-injury	45.34	0.5772 (0.4131–0.7414)	0.3016	0.5	0.8537	0.3537

## Discussion

This study monitored the dynamic changes of inflammatory factors such as NLRP3, ASC, and Caspase-1 in the Serum and CSF of patients, and found significant differences in the levels of these factors in different degrees of TBI: the more severe the injury, the higher the levels. We also found that on the 4th day post-surgery, the levels of NLRP3, ASC, and Caspase-1 in the CSF were potentially correlated with patient prognosis and could predict the prognosis of patients. Timely control of these inflammatory factors may help alleviate brain injury and promote neurological recovery. These findings provide important evidence for the clinical treatment of TBI and can help improve the overall level of medical care.

The injury mechanisms of TBI mainly include primary and secondary injuries. Primary injury is closely related to factors such as the magnitude, location, and degree of external force, which directly leads to the destruction and deformation of brain tissue. Secondary injury is the focus of clinical treatment and experimental research for TBI, involving multiple aspects such as neuroinflammatory response, oxidative stress, and cell necrosis. Secondary injury can easily lead to a "damage-inflammation-damage" vicious cycle, which further exacerbates neural injury and causes indirect damage to brain tissue. Our study combined ventricular and lumbar CSF samples to ensure adequate sample availability across TBI severity groups. However, we acknowledge potential biochemical gradients along the neuraxis that could theoretically affect biomarker distribution. Prior studies suggest ventricular CSF may reflect local inflammatory processes near the injury site, while lumbar CSF represents downstream systemic responses [25]. Notably, NLRP3 inflammasome components (Caspase-1: 45 kDa, ASC: 22 kDa) fall below the 50 kDa threshold associated with rapid CSF circulation equilibrium [26]. This molecular property, combined with standardized sampling timing (all CSF collected within 24 h post-TBI) and processing protocols (immediate centrifugation at 3000×*g*), may mitigate spatial heterogeneity in biomarker levels.

The major reasons for difficult recovery include: (1) external factors hindering neuronal re-generation and repair, and (2) intrinsic factors limiting regeneration and repair. Excitotoxicity, neuroinflammation, calcium overload, mitochondrial dysfunction, lysosomal rupture, free radical accumulation, and lipid peroxidation all affect the regeneration of neurons and recovery of neural function to varying degrees. Among these, neuroinflammation can be divided into acute and chronic forms based on its timing and duration. Both forms represent the same pathological process at different stages but differ in mechanisms and impact. Neuroinflammatory responses occur within minutes after TBI and can persist for a long time, playing a crucial role in secondary injury.

Inflammasomes play a key role in the inflammatory response, with the NLRP3 inflammasome being the most extensively studied, especially in relation to neuroinflammation and systemic inflammatory responses after TBI. As an essential component of innate immunity, the NLRP3 inflammasome plays an important role in immune responses and disease development. NLRP3, ASC, and Caspase-1 combine to form a multi-protein complex, which constitutes the inflammasome and inflammatory platform. This complex activates pro-Caspase-1 into Caspase-1 via a specific pathway, further promoting the release of pro-inflammatory cytokines such as IL-1β and IL-18, triggering an inflammatory cascade. After TBI, the mitochondrial permeability of neurons and glial cells is disrupted, releasing a large amount of ROS from damaged mitochondria into the cytoplasm, which activates the NLRP3 inflammasome and releases inflammatory factors, causing neuroinflammation. Studies have shown that inhibiting or blocking the assembly of the NLRP3 inflammasome can effectively suppress the release of downstream inflammatory factors and control the inflammation process [27].

Previous studies have shown that the levels of inflammatory factors in the serum and CSF of TBI patients are significantly elevated, and these levels increase with the decrease in Glasgow Coma Scale (GCS) scores [28]. This study also found that the levels of NLRP3, ASC, and Caspase-1 in the severe group were significantly higher than those in the mild-to-moderate group. In addition, the expression of NLRP3 inflammasome components was time-dependent in TBI [29]. The protein expression of NLRP3, ASC, and Caspase-1 began to increase 6 h after injury and continued until the 7th day. In a rodent model of penetrating ballistic-like brain injury, an acute increase in NLRP3 inflammasome components was also observed; 48 h post-injury, the number of microglial cells expressing inflammasome proteins significantly increased. These microglial cells persisted in the brain for up to 12 weeks after injury, accompanied by ongoing neurodegeneration [30]. The activation of the NLRP3 inflammasome peaked 48 h post-injury, subsequently inducing proptosis. Our study also found that NLRP3 expression peaked on the 2nd day. Initially, the activation of the inflammasome was mainly localized to neurons, then shifted to microglial cells within 24 to 48 h post-injury. Due to proptosis of neurons, inflammation in adjacent microglial cells was triggered, while astrocytes exhibited delayed inflammasome activation [31]. The temporal expression pattern of NLRP3 inflammasome components indicates that the initial cell death induced by mechanical injury rapidly increases NLRP3 to stimulate the innate immune response, which is later downregulated through the activation of protective mechanisms. In another blast-induced TBI rodent model, the activation of the NLRP3 inflammasome mediated the inflammatory response [32]. Research has also shown that thioredoxin-interacting protein (TXNIP) is a key redox-sensitive regulator of NLRP3 activity [33, 34]. Increased TXNIP expression can lead to elevated NLRP3 expression and Caspase-1 activation. Moreover, after blast injury, pro-inflammatory cytokines like IL-1β and TNF-α are significantly elevated.

Compared to wild-type mice, NLRP3 knockout mice show preserved cognitive function, less severe injury, and reduced inflammatory response in TBI [18]. Therefore, in clinical practice, we aim to implement measures during and after surgery to reduce or inhibit the expression of NLRP3 and other related inflammatory factors to improve patient prognosis. Propofol (2,6-diisopropylphenol), a widely known lipophilic intravenous anesthetic, is associated with reduced NLRP3 expression, IL-1β maturation, and oxidative stress. Additionally, the post-surgical use of telmisartan (an angiotensin receptor blocker) can re-duce brain edema and improve neurological outcomes, which is related to a reduction in NLRP3 inflammasome activation following TBI. The beneficial effects of telmisartan are achieved by inhibiting the release of IL-18 and IL-1β in an NLRP3-dependent manner [17]. Mangiferin (1,3,6,7-tetrahydroxyflavone-C2-β-D-glucoside), a compound from traditional Chinese medicine, can alleviate brain injury by inhibiting the ROS-TXNIP-NLRP3 inflammasome pathway. Recent studies have confirmed the role of oxidative stress in mediating NLRP3 inflammasome activation [35]. NOX-2, a component of NADPH oxidase and a major ROS inducer, when deficient, weakens the activation of NLRP3 inflammasome post-TBI and is associated with reduced interaction between TXNIP and NLRP3. Similarly, hyperbaric oxygen therapy can alleviate neuroinflammation and brain edema, while improving motor function [36]. However, the effect of hyperbaric oxygen therapy on NLRP3 remains controversial, as it can induce oxidative stress, which is known to activate the NLRP3 inflammasome [16, 37, 38].

Although CSF biomarkers show better performance in TBI patients, routine measurement is limited by the invasiveness of CSF sampling, which requires lumbar puncture. This procedure carries risks and discomfort for patients, making it less feasible for widespread clinical use. To overcome this, future research could focus on developing non-invasive alternatives, such as blood-based biomarkers, that correlate with CSF markers, enabling more accessible and practical diagnostic tools for TBI.

Therefore, how to more rationally reduce the levels of NLRP3-related inflammasomes is an important direction for our clinical research.

This study has several limitations that warrant consideration. First, the relatively small sample size may reduce the statistical power to detect subtle biomarker changes. Second, our observation window was limited to the first four days post-injury; extended monitoring of NLRP3, ASC, and Caspase-1 dynamics could enhance prognostic insights. Third, although combining ventricular and lumbar CSF samples improved cohort representation, potential spatial gradients along the neuraxis might theoretically influence biomarker levels. While standardized protocols (uniform processing within 30 min and 3000×*g* centrifugation) minimized technical variability, differential clearance rates between ventricular and lumbar compartments could persist as a biological confounder. Finally, the reported AUC values require cautious interpretation due to potential overfitting from data-driven cut-offs. External validation using independent cohorts is essential to confirm clinical applicability. Future studies should incorporate paired multi-site CSF sampling to dissect spatial–temporal biomarker patterns while expanding sample diversity.

## Conclusions

This study demonstrates that dynamic monitoring of NLRP3, ASC, and Caspase-1 levels in blood and CSF has significant value in assessing the severity and prognosis of TBI patients. After TBI, the levels of these factors in the serum and CSF are significantly elevated and positively correlated with injury severity. Moreover, the levels of these inflammatory factors in the CSF can effectively predict patient prognosis. Therefore, reducing the levels of related inflammatory factors after TBI and minimizing the neurotoxic substances that mediate secondary brain damage may become an effective strategy for treating TBI.

## Data Availability

No datasets were generated or analysed during the current study.
